# Carcinome métaplasique du sein avec différenciation osseuse extensive: à propos d'un cas

**DOI:** 10.11604/pamj.2013.16.36.1459

**Published:** 2013-10-01

**Authors:** Amal Benlemlih, Mouhcine Bendahou, Kaoutar Znati, Mohamed Sekkal, Sanae Chahbouni, Samia Mahmoud, Mohamed Banani, Amarti Afaf

**Affiliations:** 1 Service d'anatomie et de cytopathologie, CHU Hassan II, FES, Maroc; 2 Service de gynéco-obstétrique, CHU Hassan II, FES, Maroc

**Keywords:** Tumeur du sein, diagnostique, traitement, Breast tumor, diagnostic, treatment

## Abstract

Le carcinome métaplasique du sein est une entité rare et bien individualisé par l'OMS. Il représente moins de 1% des cancers invasifs du sein et constitue un groupe tumoral hétérogène soit purement épithélial soit à doublecontingent épithélial et mésenchymateuse. Le carcinome métaplasique avec différenciation osseuse extensive est très rare. Il représente 0.2% des carcinomes du sein. Nous rapportant un cas exceptionnel d'un carcinome métaplasique du sein avec différenciation osseuse extensive chez une patiente de 53 ans. A travers ce cas et une revue de la littérature, les caractéristiques anatomo-cliniques, radiologique, thérapeutiques et évolutives seront discutées.

## Introduction

Le carcinome métaplasique du sein est une forme rare des cancers mammaires représentant moins de 1% des cancers invasifs du sein. C'est une tumeur très hétérogène pouvant être soit purement épithéliale: tels le carcinome épidermoïde, le carcinome adénosquameux et l'adénocarcinome à différenciation fusocellulaire, soit mixte à double composante épithéliale et mésenchymateuse. Le contingent mésenchymateux pouvant être soit cartilagineux ou osseux. Le carcinome métaplasique avec différenciation osseuse extensive est très rare. Il représente 0.2% des carcinomes du sein. Nous rapportant un cas exceptionnel d'un carcinome métaplasique avec différenciation osseuse extensive chez une patiente de 53ans ayant présentée un nodule du sein gauche augmentant rapidement de taille.

## Patient et observation

Il s'agit d'une patiente âgée de 53 ans ménopausée il y'a 10 ans consultant pour une masse au niveau du sein gauche apparue 8 mois auparavant et ayant augmentée rapidement de volume.

**Figure 1:**
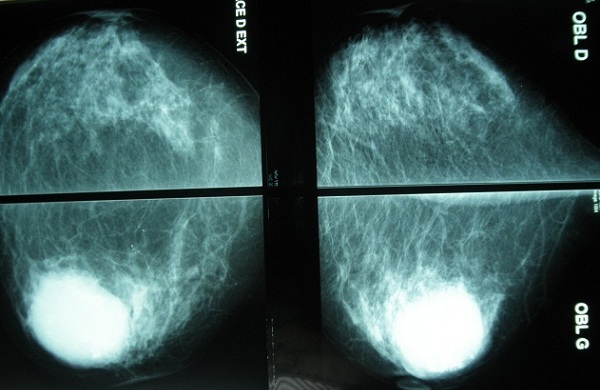
Mammographie montrant de multiples calcifications

**Figure 2:**
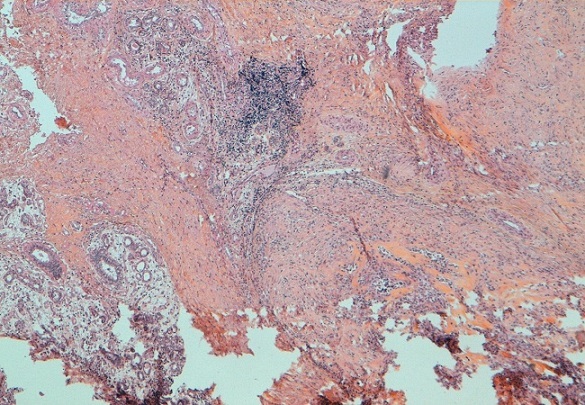
Parenchyme mammaire siège d'une prolifération tumorale à double contingent épithélial et osseux. HES x 5

**Figure 3:**
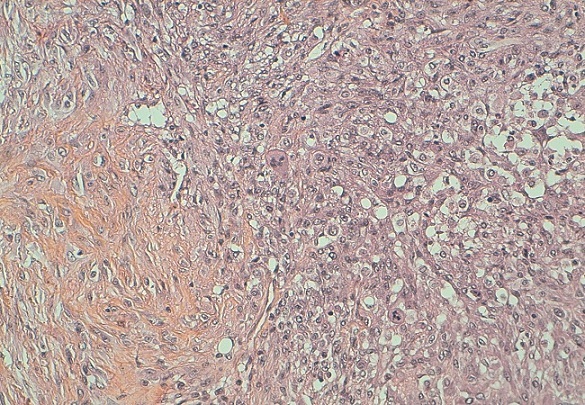
Contingent carcinomateux à droite de l'image associé à un contingent osseux à gauche de l'image. HES x 10

**Figure 4:**
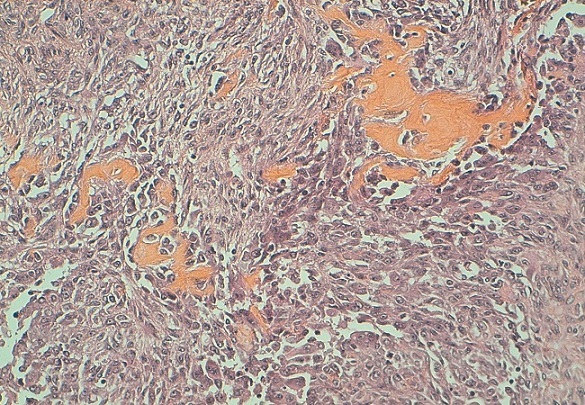
Travées osseuses immatures bordées par des cellules sarcomateuses atypiques à cytoplasme abondant éosinophile parfois clair. HES X 10

L'examen du sein gauche a révélé la présence d'une énorme tumeur de 14cm de grand axe, ferme, polylobée, mobile par rapport au 2 plans, sans signes inflammatoires en regard ni écoulement mamelonnaire avec présence d'une adénopathie axillaire homolatérale. Le couple écho-mammographie retrouvait une masse tissulaire du quadrant supéro-externe du sein gauche bien limitée comportant de multiple macro calcifications orientant soit vers un adénofibrome calcifié soit vers une tumeur phyllode calcifiée (Figure 1). L'aspect cytologique n'a pas révélé la présence de cellules néoplasiques. Une biopsie chirurgicale a donc été réalisée. L’étude histologique a retrouvée une prolifération tumorale à double contingent (Figure 2). Un contingent carcinomateux (Figure 3) fait de fines travées et de cordons cellulaires et un contingent osseux malin comportant des travées osseuses immatures bordées par des cellules sarcomateuses atypiques fusiformes ou arrondies à noyau ovalaire avec une chromatine mottée et s'entourant d'un cytoplasme abondant éosinophile (Figure 4). Un complément immunohistochimique a été réalisé montrant un phénotype basal: une absence d'expression des récepteurs hormonaux et de l'HER2 neu et une expression d'une cytokératine de bas poids moléculaire CK5/6. La patiente a bénéficié d'une mastectomie totale avec un curage ganglionnaire homo latérale n'ayant pas objectivé de métastases ganglionnaires. L’évolution est bonne avec un recul de 6 mois.

## Discussion

Le carcinome métaplasique du sein est une tumeur rare. Il représente moins de 1% des carcinomes mammaires. La différenciation osseuse est exceptionnelle. Elle se voit dans 0.2% des carcinomes du sein [1, 2]. Dans une étude récente, seulement 6 cas de carcinomes métaplasiques du sein avec différenciation osseuse extensive ont été recensés sur 81 cas de carcinomes métaplasiques [2]. A notre connaissance seulement une dizaine de cas ont été rapportés dans la littérature [2–4].

La classification OMS 2003 distingue entre la forme épithéliale pure du carcinome métaplasique comportant le carcinome épidermoïde, l'adénocarcinome avec différenciation fusocellulaire et le carcinome adéno-squameux incluant le carcinome muco-épidermoïde. La forme mixte épithéliale et mésenchymateuse comportant le carcinome canalaire avec une métaplasie cartilagineuse, le carcinome canalaire avec métaplasie osseuse et le carcinosarcome.

Le carcinome métaplasique survient chez des femmes ménopausées âgées de plus de 55 ans [4]. Dans les cas publiés de carcinomes métaplasiques avec différenciation osseuse extensive, les patientes étaient toutes ménopausées. La symptomatologie clinique est peu différente de celle observée au cours des carcinomes métaplasiques pur. Elle se manifeste par une masse palpable dont la taille varie entre 3-4cm et plus de 20cm, selon les auteurs, avec une déformation du sein et ulcération de la peau en regard. Notre patiente avait une masse de 14 cm sur 8 mois d’évolution.

Le carcinome métaplasique du sein avec différenciation osseuse extensive se manifeste à la mammographie par des calcifications [5], qui peuvent orientées à tord vers une pathologie bénigne comme un adénofibrome ou une tumeur phyllode, le cas chez notre patiente. Aussi, le carcinome métaplasique du sein avec différenciation osseuse extensive est une tumeur qui apparait d′une part plus dense que le stroma qui l'entoure [6] et d′autre part la présence de calcifications denses et spiculées au sein de la tumeur peuvent orienter vers un ostéosarcome mammaire.

Et ce n'est qu'en présence d'un radiologue avisé que ces calcifications sont distinguées du stroma ostéoide en refaisant une mammographie avec un voltage élevé pour mieux caractériser la matrice osseuse. L’échographie est non spécifique.

La ponction du sein à l'aiguille fine donne des faux négatifs dans plus de 50% des cas [7]. Les résultats obtenus par la macro biopsie ou la biopsie par mammotome semble être plus significatifs mais s'accompagnent aussi d'un risque élevé de faux négatifs. Malheureusement les macrobiopsies ne se font pas dans tous les centres, comme dans notre cas d′où la réalisation d′une biopsie chirurgicale.

Sur le plan microscopique, le carcinome métaplasique avec différenciation osseuse extensive se caractérise par la présence de deux contingents. Un contingent carcinomateux et un contingent osseux. C'est le cas de notre patiente chez qui on a objectivée une prolifération tumorale épithéliale diffuse franchement atypique associée à des cellules multinucléés rappelant les ostéoclastes et des travées osseuses safranophiles parfois bordées par des cellules malignes globuleuses. La présence de ce contingent osseux nous a posé le problème de diagnostic différentiel avec l'ostéosarcome mammaire et avec une eventuelle composante hétérogène d′une tumeur phyllode maligne. La distinction entre ces différentes tumeurs est capitale car la prise en charge et le pronostic diffère complètement.

Dans le sarcome phyllode, le stroma est franchement sarcomateux, pouvant comporter une différenciation osseuse. Et c'est l'aspect foliacé de la composante épithéliale qui permet de porté le diagnostic d'un sarcome phyllode mais qui peut manquer lors d'un prélèvement biopsique. L'ostéosarcome mammaire montre les mêmes aspects histologiques que l'ostéosarcome extra-squelettique atteignant d'autres organes. Donc, devant une biopsie l'examen histologique seul ne peut poser le diagnostic, on a recours à l'examen immunohistochimique.

Dans le carcinome métaplasique, les cellules sarcomateuses expriment la PS100 et les cytokératines de bas poids moléculaires à savoir la CK5/6, la CK14, le 34βE12. Mais cette expression est hétérogène et non diffuse pouvant donner des faux négatifs. L'anti corps anti CD34 et le bcl-2 sont positifs dans les sarcomes phyllodes.

Dans l'ostéosarcome mammaire, les cellules géantes ostéoclastiques expriment le CD68 et la composante fusocellulaire exprime la cytokératine, les récepteurs à l’‘strogène et les récepteurs à la progestérone. Alors que dans le carcinome métaplasique ces deux derniers marqueurs ne sont pas exprimés car c'est une tumeur de phénotype basal. Notre tumeur était de phénotype basal. Les cellules tumorales exprimaient d'une façon franche et diffuse l'anticorps anti CK5/6 et étaient négatifs à l'anticorps anti récepteurs à l’‘strogène et anti récepteurs à la progestérone.

Le traitement des carcinomes métaplasiques du sein n'est pas standardisé. Il est agressif d'emblée nécessitant une concertation pluridisciplinaire. Une étude rétrospective porté sur 43 patientes porteuses d'un carcinome métaplasique n'a pas montré de différence en matière de survie entre les patientes qui ont bénéficié d'un traitement chirurgical conservateur et celles traitées par une mastectomie radicale [8]. Selon cette étude, l'utilisation de la radiothérapie adjuvante semble avoir un rôle essentiel dans le contrôle des récidives locales après le traitement chirurgical conservateur [8]. Une autre étude récente portée sur 49 patientes a montré des résultats non satisfaisants de la chimiothérapie standard dans le traitement des cancers du sein de phénotype basal [9]. L'hormonothérapie n'a pas de place vue la négativité des récepteurs hormonaux et l'Hercept test est négatif ne permettant pas d'introduire une thérapeutique ciblée à base de l'herceptine.

Le pronostic est difficile à évaluer. En effet, malgrés le faite que c'est une tumeur non lymphophile, 19 à 25% des carcinomes métaplasiques avec composante chondro-osseuse présentent des métastases ganglionnaires avec 28 à 68% de survie à 5 ans [10]. Pour une taille tumorale identique, les métastases ganglionnaires sont moins fréquentes qu'en cas de carcinome canalaire infiltrant [11]. Les métastases à distance sont rares et se font par voie hématogène. Notre patiente n'avait pas de métastases ganglionnaires. Le suivi de la patiente n'a pas retrouvé de métastases à distance après un recul de seulement 6 mois.

## Conclusion

Le carcinome métaplasique du sein avec différenciation osseuse prédominante est une tumeur extrêmement rare dont le diagnostic doit être suspecté en radiologie, confirmé en histologie en cédant de l’étude immunohistochimique. Le traitement est non codifié et doit être discuté dans les staffs multidisciplinaires. Vu la rareté de ce type de carcinome son pronostic est difficile à établir.
